# Antimicrobial Resistance Profiles of Non-typhoidal *Salmonella* From Retail Meat Products in California, 2018

**DOI:** 10.3389/fmicb.2022.835699

**Published:** 2022-03-16

**Authors:** Katie Yen Lee, Edward Robert Atwill, Maurice Pitesky, Anny Huang, Kurtis Lavelle, Maribel Rickard, Marzieh Shafii, Melody Hung-Fan, Xunde Li

**Affiliations:** ^1^Department of Population Health and Reproduction, School of Veterinary Medicine, University of California, Davis, Davis, CA, United States; ^2^Western Institute for Food Safety and Security, University of California, Davis, Davis, CA, United States; ^3^Contra Costa County Public Health Laboratory, Martinez, CA, United States

**Keywords:** non-typhoidal *Salmonella enterica* (NTS), antimicrobial resistance, retail meat, phenotype, whole-genome sequencing (WGS), resistance genes, plasmid, public health surveillance

## Abstract

Non-typhoidal *Salmonella* remains a leading cause of foodborne illness in the United States, with food animal products serving as a key conduit for transmission. The emergence of antimicrobial resistance (AMR) poses an additional public health concern warranting better understanding of its epidemiology. In this study, 958 retail meat samples collected from January to December 2018 in California were tested for *Salmonella*. From multivariable logistic regression, there was a 6.47 (90% CI 2.29–18.27), 3.81 (90% CI 1.29–11.27), and 3.12 (90% CI 1.03–9.45) higher odds of contamination in samples purchased in the fall, spring, and summer than in winter months, respectively, and a 3.70 (90% CI 1.05–13.07) higher odds in ground turkey compared to pork samples. Fourteen distinct serotypes and 17 multilocus sequence types were identified among the 43 isolates recovered, with *S*. Kentucky (25.58%), *S*. Reading (18.60%), *S.* Infantis (11.63%), and *S.* Typhimurium (9.30%) comprising the top serotypes. High prevalence of resistance was observed in retail chicken isolates for streptomycin (12/23, 52.17%) and tetracycline (12/23, 52.17%), in ground turkey isolates for ampicillin (8/15, 53.34%), and in ground beef isolates for nalidixic acid (2/3, 66.67%). Fourteen (32.56%) were susceptible to all antimicrobials tested, 11 (25.58%) were resistant to one drug, and 12 (27.91%) were resistant to two drugs. The remaining six isolates (13.95%) were multidrug-resistant (MDR, ≥3 drug classes) *S*. Infantis (*n* = 4), *S*. Reading (*n* = 1), and *S*. Kentucky (*n* = 1). Whole-genome sequencing (WGS) identified 16 AMR genes and 17 plasmid replicons, including *bla*_*CTX–M–*65_ encoding ceftriaxone resistance and a D87Y mutation in *gyrA* conferring resistance to nalidixic acid and reduced susceptibility to ciprofloxacin. The IncFIB(pN55391) replicon previously identified in connection to the worldwide dissemination of pESI-like mega plasmid carriage in an emerged *S.* Infantis clone was detected in four of the six MDR isolates. Genotypes from WGS showed high concordance with phenotype with overall sensitivity and specificity of 95.31% and 100%, respectively. This study provides insight into the AMR profiles of a diversity of *Salmonella* serotypes isolated from retail meat products in California and highlights the value of routine retail food surveillance for the detection and characterization of AMR in foodborne pathogens.

## Introduction

*Salmonella enterica* is a Gram-negative, facultative anaerobic bacteria part of the *Enterobacteriaceae* family and a pathogen imparting significant global health burdens (Andino and Hanning, 2015). In the United States, non-typhoidal *Salmonella* (NTS) is a leading cause for foodborne illness and responsible for 1.35 million cases, 26,500 hospitalizations, and 420 deaths each year (Centers for Disease Control and Prevention [CDC], 2021). While infections are typically self-limiting, they can progress to systemic infection requiring clinical treatment particularly in infants, older individuals, and immunocompromised people (Foley and Lynne, 2008; Antunes et al., 2016). Furthermore, the emergence of antimicrobial-resistant *Salmonella* underscores a significant public health concern, with drug-resistant infections resulting in increased morbidities and mortalities stemming from longer duration and severity of disease and reduced treatment efficacy (Kurtz et al., 2017; Nair et al., 2018; Jajere, 2019).

While *Salmonella* is present in a large diversity of natural reservoirs, the vast majority of human salmonellosis infections arise from handling and consumption of contaminated food animal products (Callaway et al., 2008; Foley and Lynne, 2008; Andino and Hanning, 2015), attributed by poultry and livestock serving as major sources (Crump et al., 2015; Heredia and García, 2018). Emerged resistance to traditional antimicrobial agents such as ampicillin and trimethoprim-sulfamethoxazole has reduced treatment options and led to the empirical use of critically important antimicrobial drugs (Frasson et al., 2016). Fluoroquinolones (e.g., ciprofloxacin) and third-generation cephalosporins (e.g., ceftriaxone) are currently the primary treatment options for invasive salmonellosis, with the latter being an important alternative first-line treatment for pediatric infections due to the limited number of FDA-approved indications for fluoroquinolones in children (Jackson et al., 2016). Notably, antibiotics in these same drug classes are employed in veterinary medicine for treatment of food animals. While fluoroquinolone drugs have been withdrawn for use in poultry (Food and Drug Administration [FDA], 2017), enrofloxacin is currently approved for use in cattle and swine commonly for the treatment of respiratory diseases (Food and Drug Administration [FDA], 2021a). Additionally, ceftiofur—a veterinary third-generation cephalosporin drug—is used for treatment of respiratory disease in various livestock, bacterial infections in poultry, and for treatment of subclinical and clinical mastitis in dairy cattle (Food and Drug Administration [FDA], 2021a). While antimicrobial agents vastly improve health outcomes in human and veterinary medicine alike, the ubiquity and magnitude of their usage have raised concerns on the consequences of selective pressures imposed for the emergence and dissemination of multidrug-resistant (MDR) pathogens. Antimicrobial resistance (AMR) in food animals has long been implicated as a source for resistant infections in humans, and it has become a priority public health effort to monitor the persistence and dissemination of drug-resistant pathogens such as NTS and their carriage of associated genetic determinants on the farm-to-fork continuum (Karp et al., 2017).

In the United States, the National Antimicrobial Resistance Monitoring System (NARMS) monitors AMR in enteric bacteria from animals, food, and humans (Food and Drug Administration [FDA], 2020a), including retail meat, which serves as a major conduit for MDR *Salmonella*. The epidemiology of AMR is dynamic and complex; with respect to resistance in NTS, it has been observed to be variable on a multitude of factors including serotype, source, and geographic location (Hoelzer et al., 2010; Hong et al., 2016; Nyirabahizi et al., 2020; Yin et al., 2021). The objective of this study was to characterize and assess the AMR profiles of *Salmonella* isolates recovered from fresh retail chicken, ground turkey, ground beef, and pork chop samples purchased in California over a 1-year period as part of routine NARMS surveillance. This study also utilized whole-genome sequencing (WGS) with the goal to identify the diversity of AMR genes conferring drug resistance and the carriage of genetic elements of significant public health concern.

## Materials and Methods

### Study Area and Sampling

Samples in this study were collected as part of the routine NARMS retail meat testing program. From January to December 2018, a total of 958 fresh samples consisting of 478 skin-on/bone-in chicken, 240 ground turkey, 120 pork chop, and 120 ground beef were purchased from retail stores in California twice each month. Sampling locations were selected based on randomization of grocery stores by zip codes in northern (City and County of San Francisco, Contra Costa County, and Alameda County) and southern California (West Los Angeles, East Los Angeles, Ontario, and Irvine). A variety of meat types and cuts from different brands were purchased at each store. Packaging of samples in this study included modified atmospheric packaging (MAP), plastic bag, vacuum sealed, chub, paper wrapped, and plastic film packaging. Samples were transported on ice to the laboratory, refrigerated, and processed within 72 h of purchase.

### Sample Processing and Bacterial Isolation

Samples were processed per the NARMS Retail Meat Surveillance protocol. Briefly, 25 g of each sample in 250 ml buffered peptone water (Becton Dickinson, Franklin Lakes, NJ, United States) was hand massaged for 3 min or placed on a mechanical shaker at 200 rpm for 15 min. Fifty milliliters of rinsate was added to 50 ml of double-strength lactose broth (Becton Dickinson, Franklin Lakes, NJ, United States) and incubated at 35°C for 24 h. After overnight enrichment, 0.1 ml of lactose broth was transferred to 9.9 ml Rappaport-Vassiliadis R10 (RVR10) broth (Becton Dickinson, Franklin Lakes, NJ, United States) and incubated at 42°C for 16–20 h. The RVR10 enrichments were then streaked onto XLT-4 (Remel, Lenexa, KS, United States) and Hektoen Enteric (Becton Dickinson, Franklin Lakes, NJ, United States) agars and incubated at 35°C for 18–24 h. Up to two suspect *Salmonella* colonies based on colony morphology from each selective agar were then streaked to purity on blood agar plates. Isolates were shipped on dry ice to the FDA’s Center for Veterinary Medicine (CVM) for antimicrobial susceptibility testing and WGS.

### Antimicrobial Susceptibility Testing

*Salmonella* isolates were tested using a broth microdilution method for 14 antimicrobial drugs using the NARMS Gram-negative plates (Thermo Fisher Scientific, Waltham, MA, United States) per standard protocols (Food and Drug Administration [FDA], 2016). Minimum inhibitory concentration (MIC) values for each drug were used to classify isolates based on the Clinical and Laboratory Standards Institute (CLSI) guidelines. NARMS consensus interpretive criteria were used for streptomycin and azithromycin, due to unavailability of CLSI breakpoints for these two drugs (Food and Drug Administration [FDA], 2021b). Breakpoints used to classify resistant isolates for each antimicrobial drug were as follows: amoxicillin/clavulanate (≥32/16 μg/ml), ampicillin (≥32 μg/ml), azithromycin (≥32 μg/ml), cefoxitin (≥32 μg/ml), ceftriaxone (≥4 μg/ml), chloramphenicol (≥32 μg/ml), ciprofloxacin (≥1 μg/ml), gentamicin (≥16 μg/ml), meropenem (≥4 μg/ml), nalidixic acid (≥32 μg/ml), streptomycin (≥32 μg/ml), sulfisoxazole (≥512 μg/ml), tetracycline (≥16 μg/ml), and trimethoprim-sulfamethoxazole (≥4/76 μg/ml). Phenotypic resistance was presented as resistant isolates, with intermediate and susceptible isolates grouped together for analysis. Multidrug resistance was defined as resistance to ≥1 drug in ≥3 antimicrobial classes (Magiorakos et al., 2012). Due to the significance of ciprofloxacin for salmonellosis treatment and the expansion of CLSI criteria for its intermediate susceptibility range, reduced susceptibility to ciprofloxacin was also noted (≥0.12 μg/ml) (Food and Drug Administration [FDA], 2021b).

### Whole-Genome Sequencing

*Salmonella* isolates were streaked to blood agar plates, and pure colonies were extracted from overnight cultures per manufacturer’s protocol using the Qiagen DNeasy Blood & Tissue Kit (Qiagen, Valencia, CA, United States). DNA purity and quantification was assessed using the NanoDrop and Qubit fluorometer, respectively. Libraries were prepared using the Illumina Nextera XT kit per manufacturer’s protocol with quality control and quantification done on the Bioanalyzer and Qubit. Final libraries were sequenced using v2 chemistry for 2 × 250-bp paired end reads on the Illumina MiSeq platform. Sequences were demultiplexed, and adapters were removed using MiSeq Reporter. Read trimming and assembly were conducted as previously described (Tyson et al., 2015), with *de novo* assembly done using the CLC Genomics Workbench and genome annotation using NCBI’s Prokaryotic Genome Automated Pipeline (Tyson et al., 2015). Species confirmation and serotyping were determined from WGS data per the FDA NARMS Manual of Laboratory Methods (Food and Drug Administration [FDA], 2016); SeqSero1 and SeqSero2 were used for serotyping with any discrepant isolates additionally serotyped according to the Kauffmann–White scheme (Food and Drug Administration [FDA], 2016; Zhang et al., 2019). Serotypes used for analysis correspond to the final serotype determinations submitted to NCBI as attributes with whole-genome sequences, which are deposited under BioProject PRJNA292661 (Supplementary Table 1).

### Identification of Resistance Genes, Quinolone Resistance-Determining Region Mutations, and Plasmid Replicons

Resistance genes were identified from assemblies by methods previously described (Tyson et al., 2015), with Perl scripts used to identify hits (≥85% amino acid identity and ≥50% sequence length) from a reference database of compiled genes from the ResFinder (Center for Genomic Epidemiology, DTU), ARG-ANNOT (IHU Méditerranée Infection), and CARD (McMaster University) public databases. Additionally, quinolone resistance-determining region (QRDR) mutations were assessed through extraction and analysis of the *gyrA*, *gyrB*, *parC*, and *parE* genes using ClustalW in MEGA (McDermott et al., 2016). Plasmid replicons were identified using PlasmidFinder (Center for Genomic Epidemiology), with hits determined as having ≥95% identity and ≥60% coverage.

### Multilocus Sequence Typing and Minimum Spanning Trees

To assess the relationship between *Salmonella* isolates in this study, the PubMLST database^1^ was used to determine the sequence type (ST) from WGS data for each isolate based on the seven-gene legacy multilocus sequence typing (MLST) loci for *Salmonella*: *aroC*, *dnaN*, *hemD*, *hisD*, *purE*, *sucA*, and *thrA*. MLST data was then used to generate and visualize minimum spanning trees using the Global Optimal eBURST (goeBURST) algorithm (Francisco et al., 2009) with PHYLOViZ (Francisco et al., 2012).

### Data Analysis

A total of 43 *Salmonella* isolates from 41 retail meat samples were included in the analysis. Two isolates from a ground turkey and two from a ground beef sample were included due to more than one unique AMR phenotype profile recovered from each of these samples. Descriptive statistics (prevalence of *Salmonella*, distribution of covariates, and antimicrobial susceptibility testing results) and logistic regression models were conducted using SAS 9.4. The outcome binary variable for logistic regression was designated as the presence or absence of *Salmonella*, and the covariates included region of sample collection (northern and southern California), season, meat type, packaging, and label claim (conventional and reduced antibiotic use). Categorization of reduced antibiotic use included samples with packaging claims of organic and/or no antibiotic usage. All other samples with absence of organic or antibiotic claims were categorized as conventional. Univariate logistic regression was performed to determine the crude associations between the outcome and each covariate. A multivariable logistic regression model was then fitted using candidate variables with *p* < 0.25 from univariate analysis. The significance of all two-way interactions was tested, and the final model was selected based on the lowest Akaike’s Information Criterion (AIC). Variable selection for the final model was also guided by the literature where associations between *Salmonella* and factors such as meat source have been previously substantiated. Given the smaller sample sizes present in this study, a significance level of α = 0.10 was used to reduce the probability of a type II error. Genotype was considered concordant with phenotype when an isolate with phenotypic resistance to a drug had known resistance genes or mutations conferring resistance to the corresponding drug (true positive, TP) or when an isolate with phenotypic susceptibility to a drug had absence of resistance genes or mutations conferring resistance to the corresponding drug (true negative, TN). False negatives (FN) were defined as isolates that were phenotypically resistant but genotypically susceptible, and false positives (FP) were defined as isolates that were phenotypically susceptible but genotypically resistant. Sensitivity was calculated as TP/(TP + FN) and specificity as TN/(TN + FP). Matrices were created for phenotypic antimicrobial testing results and the presence/absence of resistance genes and plasmid replicons. A heatmap and hierarchical clustering were performed using the heatmap3 package in R, with dissimilarity matrices constructed using the Manhattan distance algorithm and clustered using the UPGMA method.

## Results

### Isolation of *Salmonella* From Raw Retail Meat Products in California

Out of 958 retail meat products, *Salmonella* was isolated from 41 (4.28%) samples, with the highest recovery in ground turkey (14/240, 5.83%) followed by chicken (23/478, 4.81%), ground beef (2/120, 1.67%), and pork chops (2/120, 1.67%) (Table 1).

**TABLE 1:** Prevalence and logistic regression models of risk factors for *Salmonella* in retail meat products from California.

Variable	Univariate models			Multivariable model	
		
	*Salmonella* positive *n*/*N* (%)	OR (90% CI)	*P*-value	OR (90% CI)	*P*-value
**Region**					
Northern CA	17/478 (3.56%)	0.70 (0.41–1.19)	0.272	–	–
Southern CA	24/480 (5.00%)	1.00			
**Season**					
Spring	11/240 (4.58%)	3.80 (1.29–11.20)	0.043^a^	3.81 (1.29–11.27)	0.042^b^
Summer	9/238 (3.78%)	3.11 (1.03–9.39)	0.092*^a^*	3.12 (1.03–9.45)	0.091^b^
Fall	18/240 (7.50%)	6.41 (2.27–18.07)	0.003*^a^*	6.47 (2.29–18.27)	0.003*^b^*
Winter	3/240 (1.25%)	1.00		1.00	
**Meat type**					
Chicken	23/478 (4.81%)	2.98 (0.88–10.15)	0.142*^a^*	3.01 (0.88–10.27)	0.140
Ground turkey	14/240 (5.83%)	3.66 (1.04–12.85)	0.090*^a^*	3.70 (1.05–13.07)	0.088^b^
Ground beef	2/120 (1.67%)	1.00 (0.19–5.25)	1.000	1.00 (0.19–5.27)	1.000
Pork chop	2/120 (1.67%)	1.00		1.00	
**Packaging type**					
MAP (modified atmospheric packaging)	25/554 (4.51%)	1.31 (0.61–2.80)	0.563	–	–
Plastic bag	7/95 (7.37%)	2.20 (0.86–5.64)	0.168^a^	–	–
Other (vacuum, chub, or paper)	3/137 (2.19%)	0.62 (0.19–2.01)	0.504	–	–
Plastic film	6/172 (3.49%)	1.00			
**Label claim**					
Conventional	29/624 (4.65%)	1.31 (0.74–2.33)	0.444	–	–
Reduced antibiotic claim	12/334 (3.59%)	1.00			

*^a^A p = 0.25 cut-off from univariate analysis was used for selection of candidate variables for multivariable analysis.*

*^b^Statistically significant at α = 0.10.*

### Factors Associated With *Salmonella* Contamination of Raw Retail Meat Products in California

Region of sample purchase (northern and southern CA), packaging type, and label claim were not significantly associated with the recovery of *Salmonella*, with the final multivariable logistic regression model including season and meat type as significant covariates. Odds of *Salmonella* isolation was 3.70 (90% CI 1.05–13.07) times higher in ground turkey when compared to pork chops, adjusting for season. Adjusting for meat type, samples collected in the fall, spring, and summer months had a 6.47 (90% CI 2.29–18.27), 3.81 (90% CI 1.29–11.27), and 3.12 (90% CI 1.03–9.45) times higher odds of *Salmonella* contamination compared to those collected in the winter months, respectively (Table 1).

### Distribution of *Salmonella* Serotypes and Multilocus Sequence Typing Profiles

**FIGURE 1:**
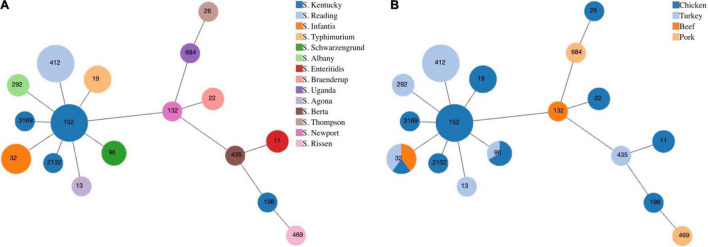
Minimum spanning tree based on multilocus sequence typing of seven housekeeping genes for *Salmonella* isolates from retail meat, by panel **(A)** serotype and **(B)** meat type. Each circle represents one sequence type and is indicated by the number in the circle. The size of each circle corresponds to the number of isolates.

From serotyping and MLST analysis, 14 distinct serotypes and 17 STs were identified (Table 2). The most frequently isolated serotypes were *S*. Kentucky (11/43, 25.58%) and *S*. Reading (8/43, 18.60%), with all *S*. Kentucky isolates recovered from chicken samples and all *S*. Reading isolates recovered from ground turkey. The remaining 12 serotypes displayed distinctive source trends, with exceptions of *S.* Infantis being recovered from three different meat types—chicken, ground turkey, and ground beef—and *S*. Schwarzengrund recovered from chicken and ground turkey (Table 2). Each serotype was associated with one ST, with the exception of *S*. Kentucky isolates, which were distributed across four different STs (Figure 1A). By source, ST32 isolates were recovered across different retail meats (Figure 1B). The greatest serotype and ST diversity was observed in isolates from chicken samples (Figure 1B), though the wide distribution of isolates overall is indicative of a high degree of diversity in genetic profiles across all *Salmonella* isolates recovered in this study (Figure 1).

**TABLE 2:** Distribution of serotypes and multilocus sequence typing patterns for *Salmonella* isolates (*n* = 43).

Serotype	Retail meat types (no. of isolates)					MLST pattern							
		
	Chicken (*n* = 23)	Ground turkey (*n* = 15)	Ground beef (*n* = 3)	Pork (*n* = 2)	Total *n*/*N* (%)	*aroC*	*dnaN*	*hemD*	*hisD*	*purE*	*sucA*	*thrA*	ST
*S.* Agona	0	1	0	0	1/43 (2.33%)	3	3	7	4	3	3	7	13
*S.* Albany	0	2	0	0	2/43 (4.65%)	104	100	54	78	104	9	48	292
*S.* Berta	0	1	0	0	1/43 (2.33%)	2	2	3	124	2	2	6	435
*S.* Braenderup	2	0	0	0	2/43 (4.65%)	12	2	15	14	11	14	16	22
*S.* Enteritidis	2	0	0	0	2/43 (4.65%)	5	2	3	7	6	6	11	11
*S.* Infantis	1	2	2	0	5/43 (11.63%)	17	18	22	17	5	21	19	32
*S.* Kentucky	8	0	0	0	11/43 (25.58%)	62	53	54	60	5	53	54	152
	1	0	0	0		62	53	54	60	636	53	54	3,169
	1	0	0	0		76	14	3	77	64	64	67	198
	1	0	0	0		62	53	54	60	508	53	54	2,132
*S.* Newport	0	0	1	0	1/43 (2.33%)	2	57	15	14	15	20	12	132
*S*. Reading	0	8	0	0	8/43 (18.60%)	11	10	25	13	10	58	4	412
*S.* Rissen	0	0	0	1	1/43 (2.33%)	92	107	79	156	64	151	87	469
*S.* Schwarzengrund	2	1	0	0	3/43 (6.98%)	43	47	49	49	41	15	3	96
*S.* Thompson	1	0	0	0	1/43 (2.33%)	14	13	18	12	14	18	1	26
*S.* Typhimurium	4	0	0	0	4/43 (9.30%)	10	7	12	9	5	9	2	19
*S.* Uganda	0	0	0	1	1/43 (2.33%)	147	13	15	123	15	19	17	684
													

### Phenotypic Antimicrobial Resistance Profiles

All 43 of the *Salmonella* isolates in this study were susceptible to azithromycin, meropenem, and trimethoprim-sulfamethoxazole; 32.56% (14/43) of isolates were susceptible to all antimicrobials tested, 25.58% (11/43) were resistant to one drug, 27.91% (12/43) to two drugs, and 13.95% (6/43) to three or more antimicrobial drugs tested. The highest resistance was observed for tetracycline (17/43, 39.53%), followed by streptomycin (15/43, 34.89%) and ampicillin (10/43, 23.26%). Resistance to aminoglycoside drugs—gentamicin and streptomycin—were observed in chicken and ground turkey isolates, with over half of the isolates from chicken samples displaying resistance to streptomycin (12/23, 52.17%). Resistance to sulfonamides—sulfisoxazole—was only detected in chicken and ground turkey isolates (Table 3).

**TABLE 3:** Percentage of *Salmonella* isolates resistant to antimicrobials from phenotypic susceptibility testing, by retail meat type.

CLSI class	Antimicrobial rank*^a^*	Antimicrobial agent	Chicken (*n* = 23)	Ground turkey (*n* = 15)*^b^*	Ground beef (*n* = 3)*^c^*	Pork (*n* = 2)	Total *n*/*N* (%)
Aminoglycosides	1	GEN	1 (4.35%)	2 **(13.33%)**	0	0	3/43 (6.88%)
	1	STR	12 (**52.17%)**	3 (20.00%)	0	0	15/43 (34.89%)
B-lactam combination agents	1	AMC	1 (4.35%)	0	0	0	1/43 (2.33%)
Cephems	2	FOX	1 **(4.35%)**	0	0	0	1/43 (2.33%)
	1	AXO	1 (4.35%)	2 (13.33%)	1 **(33.33%)**	0	4/43 (9.30%)
Folate pathway antagonists	2	FIS	5 **(21.74%)**	2 (13.33%)	0	0	7/43 (16.28%)
	2	COT	0	0	0	0	0/43 (0%)
Macrolides	1	AZI	0	0	0	0	0/43 (0%)
Penems	1	MER	0	0	0	0	0/43 (0%)
Penicillins	1	AMP	1 (4.35%)	8 **(53.34%)**	1 (33.33%)	0	10/43 (23.26%)
Phenicols	2	CHL	1 **(4.35%)**	0	0	0	1/43 (2.33%)
Quinolones	1	NAL	1 (4.35%)	2 (13.33%)	2 **(66.67%)**	0	5/43 (11.63%)
	1	CIP*^d^*	2 (8.70%)*^d^*	2 (13.33%)^d^	2 **(66.67%)***^d^*	0	6/43 (13.95%)*^d^*
Tetracyclines	2	TET	12 **(52.17%)**	3 (20.00%)	1 (33.33%)	1 (50.0)	17/43 (39.53%)

*Meat type with the highest percentage of resistant isolates for each drug is rendered in bold.*

*^a^Rank based on the WHO categorization of antimicrobials of critical importance to human medicine.*

*^b^Two isolates with different phenotypic profiles were included from one ground turkey sample.*

*^c^Two isolates with different phenotypic profiles were included from one ground beef sample.*

*^d^Results presented for ciprofloxacin (CIP) are for intermediate susceptibility.*

*GEN, gentamicin; STR, streptomycin; AMC, amoxicillin-clavulanic acid; FOX, cefoxitin; AXO, ceftriaxone; FIS, sulfisoxazole; COT, trimethoprim-sulfamethoxazole; AZI, azithromycin; MER, meropenem; AMP, ampicillin; CHL, chloramphenicol; NAL, nalidixic acid; CIP, ciprofloxacin; TET, tetracycline.*

Resistance to all three beta-lactam combination agent drugs tested (amoxicillin-clavulanic acid, cefoxitin, and ceftriaxone) was observed in one chicken isolate. This was a *S*. Kentucky isolate resistant to beta-lactam/combination, aminoglycoside, and penicillin drug classes and one of the six MDR isolates identified in this study. The other five MDR isolates included four *S.* Infantis isolates recovered from a ground turkey (*n* = 2), a ground beef (*n* = 1), and a chicken (*n* = 1) sample and a *S*. Reading isolate from a ground turkey sample. Notably, four of the six MDR isolates (all *S.* Infantis) displayed resistance to nalidixic acid and reduced susceptibility to ciprofloxacin, and resistance to ceftriaxone was only observed in MDR isolates (Tables 3, 4).

**TABLE 4:** Antimicrobial resistance (AMR) patterns of *Salmonella* serotypes resistant to one or more antimicrobial drugs from retail meat in California, 2018*^a–d^*.

Serotype		Antimicrobial pattern (no. of isolates)

Name	No. of isolates *n*/*N* (%)*^b^*	
*S*. Albany	2/43 (4.65%)	GEN (*n* = 1)
		GEN-STR (*n* = 1)
*S.* Infantis	5/43 (11.63%)	NAL-CIP*^c^* (*n* = 1)*^e^*
		**AMP-AXO-NAL-TET-CIP*^c^* (*n* = 2)** *^e,f^*
		**AMP-AXO-NAL-STR-FIS-TET-CIP*^c^* (*n* = 1)** *^f^*
		**CHL-GEN-NAL-STR-FIS-TET-CIP*^c^* (*n* = 1)**
*S*. Kentucky	9/43 (20.93%)	STR (*n* = 1)
		STR-TET (*n* = 6)
		STR-TET-CIP*^c^* (*n* = 1)
		**AMC-AMP-FOX-AXO-STR (*n* = 1)**
*S*. Reading	6/43 (13.95%)	AMP (*n* = 5)
		**AMP-STR-FIS-TET (*n* = 1)**
*S*. Rissen	1/43 (2.33%)	TET (*n* = 1)
*S*. Schwarzengrund	2/43 (4.65%)	STR (*n* = 2)
*S*. Typhimurium	4/43 (9.30%)	FIS-TET (*n* = 4)

Total	29/43 (67.44%)	–

*Multidrug-resistant isolates are rendered in bold.*

*^a^Isolates from the following serotypes not listed were susceptible to all 14 antibiotics tested: S. Agona (n = 1), S. Berta (n = 1), S. Braenderup (n = 2), S. Enteritidis (n = 2), S. Kentucky (n = 2), S. Newport (n = 1), S. Reading (n = 2), S. Schwarzengrund (n = 1), S. Thompson (n = 1), and S. Uganda (n = 1).*

*^b^Percentages calculated as number of isolates resistant to one or more antimicrobial drugs to the total number of Salmonella isolates in the study.*

*^c^Ciprofloxacin (CIP) in antimicrobial patterns indicates intermediate susceptibility.*

*^d^Multidrug resistance (MDR) was defined as resistance to ≥1 drug in ≥3 antimicrobial classes.*

*^e^Isolates were recovered from the same ground beef sample.*

*^f^Isolates were recovered from the same ground turkey sample.*

*GEN, gentamicin; STR, streptomycin; AMC, amoxicillin-clavulanic acid; FOX, cefoxitin; AXO, ceftriaxone; FIS, sulfisoxazole; COT, trimethoprim-sulfamethoxazole; AZI, azithromycin; MER, meropenem; AMP, ampicillin; CHL, chloramphenicol; NAL, nalidixic acid; CIP, ciprofloxacin; TET, tetracycline.*

Five distinct antibiogram profiles were observed in MDR isolates, with two *S.* Infantis isolates displaying the tetra-resistant pattern ampicillin, ceftriaxone, tetracycline, and nalidixic acid in addition to reduced susceptibility to ciprofloxacin. In non-MDR isolates, the most common resistance patterns observed were streptomycin and tetracycline (STR-TET, *n* = 6) followed by sulfisoxazole and tetracycline (FIS-TET, *n* = 4) (Table 4).

### Detection of Antimicrobial Resistance Genes and Plasmid Replicons

**FIGURE 2:**
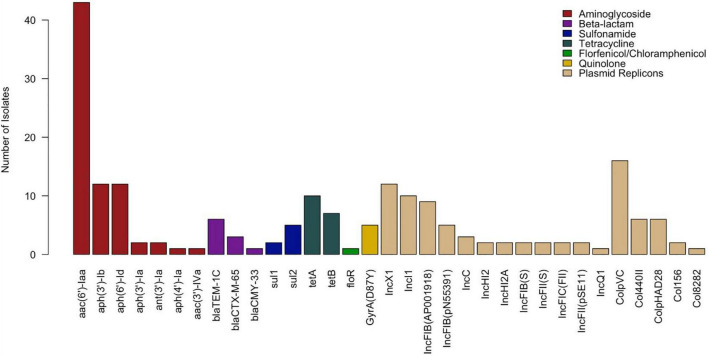
Distribution of antimicrobial resistance genes and plasmid replicons detected in *Salmonella* isolates (*n* = 43).

Among the 43 *Salmonella* isolates, 16 distinct antimicrobial genes and 17 plasmid replicons were identified. Resistance genes encoding all three types of aminoglycoside-modifying enzymes (AMEs)—acetyltransferases *(aac(6’)-Iaa* and *aac(3’)-IVa)*, nucleotidyltransferases *(ant(3’)-Ia)*, and phosphotransferases *(aph(3’)-Ib*, *aph(6’)-Id, aph(3’)-Ia*, and *aph(4’)-Ia)*—were detected in this study. Beta-lactamase genes detected included *bla*_*TEM–*1*C*_ and extended spectrum beta-lactamase (ESBL) *bla*_*CTX–M–*65_ from class A and AmpC-type *bla*_*CMY–*33_ from class C. Quinolone resistance-mediating genes detected included a mutation of the QRDR of *gyrA* (D87Y). No plasmid-mediated quinolone resistance (PMQR) genes were detected. Other genes detected included those encoding resistance to tetracycline (*tetA* and *tetB*), sulfonamide (*sul1* and *sul2*), and florfenicol-chloramphenicol (*floR*). Eighty-six percent (37/43) of isolates carried at least one plasmid replicon, 55.8% (24/43) carried two or more, and 39.5% (17/43) carried three or more. The most commonly detected plasmids were ColpVC (16/43, 37.21%), IncX1 (12/43, 27.91%), IncI1 (10/43, 23.26%), and IncFIB(AP001918) (9/43, 20.93%). The distribution of all AMR genes and plasmid replicons is presented in Figure 2.

### Correlation Between Genotype and Phenotype

In this study, resistance genes identified from WGS correlated with phenotypic testing results with an overall sensitivity and specificity of 95.31% and 100%, respectively. Sensitivity was not calculated for azithromycin, meropenem, trimethoprim-sulfamethoxazole, and ciprofloxacin, due to the absence of resistant isolates to these drugs. Discrepancies were present in aminoglycosides, in which two of the three gentamicin-resistant isolates and one of the 15 streptomycin-resistant isolates did not carry resistance-conferring genes, resulting in a sensitivity of 33.33% and 93.33% respectively, for these drugs (Table 5).

**TABLE 5:** Evaluation of genotype prediction of phenotypic resistance.

CLSI class	Antimicrobial agent	Phenotype: susceptible (no. of isolates)		Phenotype: resistant (no. of isolates)		Sensitivity*^b^* (%)	Specificity*^c^* (%)
			
		Genotype: resistant (FP)*^a^*	Genotype: susceptible (TN)*^a^*	Genotype: resistant (TP)*^a^*	Genotype: susceptible (FN)*^a^*		
Aminoglycosides	GEN	0	40	1	2	33.33	100
	STR	0	28	14	1	93.33	100
B-lactam combination agents	AMC	0	42	1	0	100	100
Cephems	FOX	0	42	1	0	100	100
	AXO	0	39	4	0	100	100
Folate pathway antagonists	FIS	0	36	7	0	100	100
	COT	0	43	0	0	N/A*^d^*	100
Macrolides	AZI	0	43	0	0	N/A*^d^*	100
Penems	MER	0	43	0	0	N/A*^d^*	100
Penicillins	AMP	0	33	10	0	100	100
Phenicols	CHL	0	42	1	0	100	100
Quinolones	NAL	0	38	5	0	100	100
	CIP	0	43	0	0	N/A*^d^*	100
Tetracyclines	TET	0	26	17	0	100	100

Overall		0	538	61	3	95.31	100

*^a^FP, false positive; TN, true negative; TP, true positive; FN, false negative. ^b^Sensitivity was calculated as TP/(TP + FN). ^c^Specificity was calculated as TN/(TN + FP). ^d^Sensitivity could not be calculated because none of the isolates were resistant to these drugs. GEN, gentamicin; STR, streptomycin; AMC, amoxicillin-clavulanic acid; FOX, cefoxitin; AXO, ceftriaxone; FIS, sulfisoxazole; COT, trimethoprim-sulfamethoxazole; AZI, azithromycin; MER, meropenem; AMP, ampicillin; CHL, chloramphenicol; NAL, nalidixic acid; CIP, ciprofloxacin; TET, tetracycline.*

### Hierarchical Clustering of *Salmonella* Isolates by Phenotype, Genotype, and Plasmid Replicon Profiles

**FIGURE 3:**
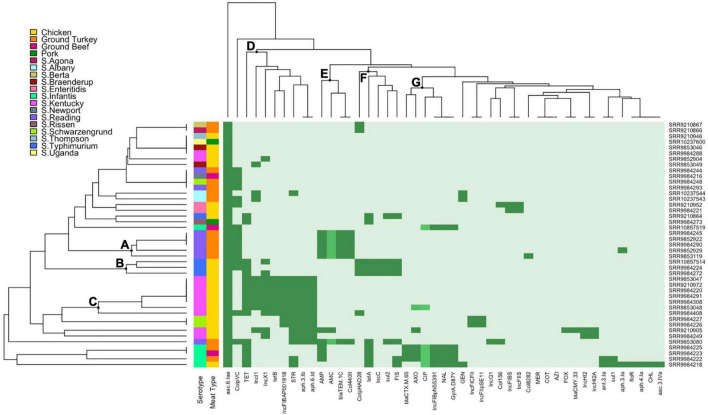
Heatmap and hierarchical clustering of *Salmonella* isolates by phenotypic antimicrobial resistance (AMR), antimicrobial genes, and plasmid replicons. Dark green represents resistant phenotype, presence of gene, or presence of plasmid replicon. Medium green indicates reduced susceptibility for ciprofloxacin or intermediate resistance for amoxicillin-clavulanic acid and ceftriaxone. Light green indicates susceptible phenotype, absence of gene, or absence of plasmid replicon. Letters (A–G) indicate the main clusters discussed in the text. GEN, gentamicin; STR, streptomycin; AMC, amoxicillin-clavulanic acid; FOX, cefoxitin; AXO, ceftriaxone; FIS, sulfisoxazole; COT, trimethoprim-sulfamethoxazole; AZI, azithromycin; MER, meropenem; AMP, ampicillin; CHL, chloramphenicol; NAL, nalidixic acid; CIP, ciprofloxacin; TET, tetracycline.

Hierarchical clustering of *Salmonella* isolates depicts the co-occurrence of specific AMR profiles and plasmid replicon types by serotype and source of isolates. The row dendrogram produced three notable clusters based on isolate-specific profiles. Cluster A corresponds to over half of the *S*. Reading isolates in this study, with the five isolates in this cluster all ampicillin resistant through carriage of a *bla*_*TEM–*1*C*_ gene and displaying intermediate resistance to amoxicillin-clavulanic acid. Three of the four *S*. Typhimurium isolates in this study were presented in cluster B, sharing chicken source and phenotypic resistance to sulfisoxazole and tetracycline conferred through *sul2* and *tetA* genes, respectively. Cluster C included the *S*. Kentucky isolates resistant to streptomycin and tetracycline (*n* = 7), with resistance conferred through *aph(3’)-Ib* (*strA*) and *aph(6’)-Id* (*strB*), and the other tetracycline gene detected in this study, *tetB* (Figure 3).

Main clusters from the column dendrogram depict the co-occurrence of phenotypes, AMR genes, and plasmid replicons. IncI1, IncX1, and IncFIB(AP001918) replicons were detected primarily in tetracycline (*tetB*) and streptomycin (*aph(6’)-Id* and *aph(3’)-Ib*) resistant *S.* Kentucky isolates (cluster D). The Col440II and ColpVC replicons were detected in ampicillin-resistant and amoxicillin-clavulanic acid intermediate-resistant *S*. Reading isolates with carriage of *bla*_*TEM–*1*C*_ (cluster E). IncC and ColpHAD28 replicons were detected in tetracycline (*tetA*) and sulfisoxazole-resistant (*sul2*) *S*. Typhimurium isolates (cluster F). Lastly, the IncFIB(pN55391) plasmid replicon was detected in MDR *S.* Infantis isolates exhibiting third-generation cephalosporin (ceftriaxone, *bla*_*CTX–M–*65_) and quinolone (nalidixic acid, *gyrA* mutation) resistance and fluoroquinolone reduced susceptibility (ciprofloxacin, *gyrA* mutation) (cluster G).

## Discussion

Despite improvements to regulatory and farm-to-fork practices in biosecurity, animal husbandry, and HACCP standards, NTS remains a leading pathogen responsible for foodborne illness in the United States. Control and elimination of *Salmonella* in retail meat products are challenging, with food animals serving as perpetual vectors and reservoirs through clinical disease and as asymptomatic carriers, contamination of farm environments, and vertical and horizontal transmission (Jajere, 2019). Though a high proportion of *Salmonella* isolates from food animal origins are pan susceptible, the presence of multiple drug-resistant phenotypes in these isolates remains comparably high relative to that in human clinical isolates, where resistance has remained relatively stable in the past decade (Food and Drug Administration [FDA], 2020b). The persisting recurrence of *Salmonella* outbreaks traced to food animal products and evidence for the emergence, evolution, and dissemination of drug-resistant strains emphasize the importance of active surveillance of AMR in foodborne pathogens. Here, we provide an insight on the presence of *Salmonella* in retail meat products purchased in California and the corresponding AMR phenotypic and genotypic profiles.

### *Salmonella* Contamination of Retail Meat Products

In this study, we observed similar frequencies of *Salmonella* contamination in fresh retail meat products purchased in California (4.3%) compared to the national average from routine NARMS surveillance in the same year (4.0%, 2018) (Food and Drug Administration [FDA], 2020b). A comparison of prevalence findings from different studies should be assessed with caution, as variability in sample collection methods, location, time, and isolation protocols may affect results. Nevertheless, our results here are consistent with previous findings of poultry products being more frequently contaminated with *Salmonella* than other meat types (Jørgensen et al., 2002; Hyeon et al., 2011; Kim et al., 2012; Li et al., 2020) due to the high-frequency colonization and carriage of *Salmonella* in the microbiome of healthy poultry animals (Antunes et al., 2016). The highest recovery of *Salmonella* in our study was in ground turkey (14/240, 5.83%) followed by chicken (23/478, 4.81%) samples, likely due to ground poultry counterparts requiring additional processing steps, which increase opportunities for cross-contamination.

The highest recovery of *Salmonella* from retail meats was observed in fall months in this study. While a previous study also found greater prevalence in fall months (Xu et al., 2020), overall findings have been inconsistent with respect to seasonality trends (Zhao et al., 2001; Zdragas et al., 2012; Wang et al., 2014; Erickson et al., 2018). It has been suggested that temporal trends may occur by year as opposed to season (Sivaramalingam et al., 2013) and that the discordances in observed seasonal contamination of *Salmonella* in food products are likely confounded by other contributing factors from production, processing, and distribution processes that serve as primary drivers of pathogen contamination and proliferation. From a food safety perspective, this contrasts with the seasonality trends observed in incidences of human salmonellosis, which has been associated with factors such as temperature and oscillations in human activity (e.g., increased consumption of meat products during certain times of the year) rather than differences in the frequency of retail meat contamination itself (Ravel et al., 2010).

### Distribution of *Salmonella* Serotypes and Antimicrobial Resistance Profiles

An important facet to *Salmonella* epidemiology is the fluctuating significance, distribution, and AMR profiles of serotypes over time. *Salmonella* serotypes by nature display host specificity and varied pathogenicity for human and animal hosts depending on their degree of adaptation (Jajere, 2019). Common serotypes that have been associated with foodborne disease have been broad-spectrum host adapted such as *S*. Enteritidis, *S*. Typhimurium, and the monophasic *S*. Typhimurium variant *S*. 1,4,[5],12:i:- (Yang et al., 2015; Antunes et al., 2016; Jajere, 2019; Mandilara et al., 2021).

The two *S*. Enteritidis and four *S*. Typhimurium isolates recovered in this study were all from retail chicken products, of which both *S*. Enteritidis isolates were susceptible to all 14 antibiotics tested and the four *S*. Typhimurium isolates displayed resistance to two drugs, tetracycline and sulfisoxazole. Tetracycline and sulfonamides are two major classes of antibiotics that have been conventionally utilized for prophylactic and therapeutic treatment of food animals (Granados-Chinchilla and Rodríguez, 2017). Tetracycline is a broad-spectrum bacteriostatic agent that was traditionally widely administered to poultry flocks through drinking water and feed (Chopra and Roberts, 2001). As of January 2017, its application in feed has been limited to therapeutic use through a requirement of the Veterinary Feed Directive (Granados-Chinchilla and Rodríguez, 2017). Despite this restriction, the highest frequency of resistance in our study was to tetracycline (17/43, 39.5%), driven by the proportionately large number of resistant chicken isolates (12/23, 52.2%). We observe a similar trend in streptomycin (15/43, 34.9%) due to the high level of resistance across all serotypes from chicken isolates (12/23, 52.2%). Streptomycin is another drug that is historically used in food-producing animals and serves as both an indicator for aminoglycoside resistance in the food supply chain (McDermott et al., 2016; Wang et al., 2019) and an epidemiologic marker for presence of penta resistance to ampicillin, chloramphenicol, streptomycin, sulfonamide, and tetracycline (ACSSuT), a pattern observed in widely disseminated virulent and MDR strains like *S*. Typhimurium DT104 and U302 (Yu et al., 2008). The third most frequent resistant drug observed in our study was ampicillin (10/43, 23.3%) attributed by ground turkey isolates (8/15, 53.3%), which is consistent to the routinely higher level of ampicillin resistance detected through NARMS surveillance in retail turkey isolates (35.5%, 2018) relative to those from retail chicken, beef, and pork (Food and Drug Administration [FDA], 2020b,2021c; Singer et al., 2020). Lastly, the detection of four MDR *S.* Infantis isolates from retail chicken, ground turkey, and ground beef products in our study mirrors NARMS surveillance findings from the last few years in which the rise in MDR *Salmonella* from retail meats is attributed by a marked increase in MDR *S.* Infantis superseding other leading resistant serotypes (Food and Drug Administration [FDA], 2021c; Tyson et al., 2021).

### Whole-Genome Sequencing for Prediction of Antimicrobial Resistance

Increasing affordability and improved turnaround time for WGS have vastly improved the resolution of foodborne bacteria profiling and allowed for its integration in routine surveillance efforts as done here in this study. In particular, its utility for identification of resistance mechanisms provides the genotypic basis for *in silico* predictions of phenotypic resistance including resistance for drugs that are not included in routine testing (McDermott et al., 2016; Su et al., 2019; NIHR Global Health Research Unit on Genomic Surveillance of AMR, 2020). Despite the small number of isolates in this study and the large proportion that are susceptible, our findings here affirm results from previous studies that demonstrated the robust capacity of WGS for prediction of phenotypic resistance in *Salmonella* and other bacterial species (Tyson et al., 2015, 2018; McDermott et al., 2016; Neuert et al., 2018). For the 43 *Salmonella* isolates here, WGS data predicted phenotypic results with an overall sensitivity and specificity of 95.31% and 100%, respectively.

The three discordant isolates resulting in lowered sensitivity were observed for gentamicin and streptomycin, attributed by phenotypic resistance in the absence of detected resistance genes. One potential explanation is that genes may not have been present when a colony was sequenced at a different time from when phenotypic testing was performed, but moreover, the imperfect correlation observed here presents a few important considerations. First, concordant genotypic prediction of phenotypic resistance is reliant on the recommended breakpoints used for classification of isolates. False classification of phenotypic results can occur when MIC values fall just below or above a breakpoint and/or in instances where interpretation of MICs is based on alternative guidelines like NARMS consensus interpretative criteria in the absence of available CLSI criteria. This is a likely explanation for the discordant streptomycin results observed here and previous studies (Tyson et al., 2015, 2018; McDermott et al., 2016; Neuert et al., 2018; Pornsukarom et al., 2018), as streptomycin is a drug traditionally used for food animals but not in the treatment of enteric infections and therefore lacks defined CLSI clinical breakpoints (McDermott et al., 2016; Wang et al., 2019). Secondly, in instances where interpretative criteria are available, alternative use of different breakpoints for classification can also affect concordance of WGS results with phenotype. For instance, specificity for ciprofloxacin in this study was fully concordant only when using CLSI breakpoints for resistance (≥1 μg/ml). The CLSI breakpoint for reduced ciprofloxacin susceptibility (≥0.12 μg/ml) is currently used as an alternative criterion for classification of resistance to capture emerging fluoroquinolone resistance (Food and Drug Administration [FDA], 2021b). Traditionally, resistance to fluoroquinolones is conferred through one or more chromosomal mechanisms mediated by mutations in the QRDRs of target enzymes DNA gyrase (*gyrA* and *gyrB*) and topoisomerase IV (*parC* and *parE*) (Hooper and Jacoby, 2016). For ciprofloxacin, resistance is observed to be conferred in combinations of mutations within both *gyrA* and *parC* (Redgrave et al., 2014; Neuert et al., 2018). Five isolates in this study exhibited reduced susceptibility, but classification of these isolates as resistant would have resulted in lowered specificity—88.37% (38/43) instead of 100%—as these isolates only carried a single *gyrA* mutation. Lastly, WGS predictions of phenotypic resistance are as robust as our ability to identify the genetic determinants that confer resistance. This highlights the impact of reference database(s) selection, importance of active curation and inclusion of novel genes to ensure database comprehensiveness, and an unavoidable caveat of relying on WGS approaches when unknown resistance mechanisms cannot be detected.

### Co-occurrence of Resistance Profiles and Plasmid Replicons by Serotype and Source

Although the significance of the associations between serotypes and the presence/absence of AMR genes and plasmid replicons could not be assessed due to the small number of isolates recovered for each serotype, results here are congruent with other studies that have reported co-occurrence of certain plasmid(s) and AMR genes with serotype (Mather et al., 2013; Pornsukarom et al., 2018; Zhao et al., 2020). It should be noted that while the presence of a plasmid replicon is likely indicative of the corresponding plasmid type, it is possible that the replicon type may have been chromosomally integrated or co-integrated on plasmids with multiple replicons (Johnson et al., 2007; McMillan et al., 2020). Such occurrences are considered rare, and the detection of plasmid replicons as done here presents a quick and efficient way to screen for the presence of putative plasmids.

In this study, we identified several plasmid replicons previously detected in *Salmonella* that are associated with resistance. IncX1 and IncI1 are two incompatibility groups frequently distributed in *Enterobacteriaceae*, with the latter frequently found in *Salmonella* from food animal sources (Kaldhone et al., 2019), as evident through the *S*. Kentucky isolates from chicken samples in this study. IncC (formerly grouped as IncA/C) plasmids are also frequently prevalent in pathogenic *Enterobacteriaceae* and are associated with *bla*_*CMY*_ and *bla*_*NDM*_ genes (Hancock et al., 2017; Ambrose et al., 2018), though here we observed their co-occurrence with *sul2* and *tetA* genes in *S*. Typhimurium isolates from chicken. In *S*. Reading turkey isolates from this study, we detected the Col440II replicon and *bla*_*TEM–*1*C*_. Carriage of *bla*_*TEM–*1*C*_ on Col440II was first detected in a *S*. Hadar turkey isolate in 2007, where thereafter detection of this plasmid with *bla*_*TEM–*1*C*_ was reported in *S*. Reading in 2014, also from a turkey source (Miller et al., 2020). Recently, *S*. Reading isolates with this plasmid were identified in an emerged clade linked to United States and Canadian outbreaks from live turkeys and raw turkey products, including one which occurred during our 2018 study period (November 2017 to March 2019) (Centers for Disease Control and Prevention [CDC], 2019; Hassan et al., 2019; Miller et al., 2020). The genomic investigation of isolates from clinical, meat product, environmental, and animal sources from Miller et al. indicated that a novel clone of *S*. Reading emerged and disseminated across North America in parallel to expansion of commercial turkey production, likely through vertical transmission from a common source (Miller et al., 2020). Detection of turkey isolates with the distinguishing carriage of Col440II and *bla*_*TEM–*1*C*_ gene here supports their findings and highlights the value of integrated surveillance in detection and elucidation of emerging microbial hazards in the food supply chain.

Another public health concern to note from this study is the detection of the IncFIB(pN55391) plasmid replicon among four MDR *S.* Infantis isolates. The IncFIB(pN55391) plasmid was first detected in MDR, ESBL-producing *S.* Infantis strains in Israel, where thereafter rapid clonal expansion resulted in its worldwide dissemination (Franco et al., 2015; Hindermann et al., 2017; Alba et al., 2020; García-Soto et al., 2020). To date, MDR ESBL-producing *S.* Infantis has been reported across the United States in humans, food animals, and—as evidenced here and in previous studies—retail meats (Tate et al., 2017; Brown et al., 2018; Alba et al., 2020; M’ikanatha et al., 2021; Tyson et al., 2021). In the past few years, NARMS surveillance has reported a rise in resistant *Salmonella* isolates from retail meats, which is attributed to increasing numbers of MDR *S.* Infantis (Tyson et al., 2021). Dissemination of *S.* Infantis with ESBL carriage on a large conjugative plasmid as indicated by four of the five isolates carrying *bla*_*CTX–M–*65_ in this study is concerning due to its potential to disseminate resistance genetic elements to other pathogens and the challenges in treating infections exhibiting resistance to penicillins, extended-spectrum cephalosporins, monobactams, and other drugs conferred through MDR status (Tate et al., 2017; M’ikanatha et al., 2021).

## Conclusion

Despite the relatively low frequency of *Salmonella* contamination observed in retail meat products in this study, the diversity of serotypes and AMR profiles present in the isolates recovered highlights the risk of retail meat products as reservoirs and conduits for drug-resistant NTS. Findings here also demonstrate the complementary role of WGS with phenotypic testing for the high-resolution profiling of foodborne pathogens. Moreover, this study sheds light on the importance of surveillance for the assessment of emerging and circulating AMR hazards and the need to continue these efforts to best guide intervention measures for AMR mitigation across farm-to-fork interfaces.

## Data Availability Statement

The datasets presented in this study can be found in online repositories. The names of the repository/repositories and accession number(s) can be found in the article/Supplementary Material.

## Author Contributions

KLe, EA, MP, and XL conceptualized the study. KLe, AH, MR, MS, and MH-F performed the laboratory analysis. KLe, AH, KLa, MR, MS, and MH-F curated the data. KLe analyzed the data and contributed in the writing—original draft preparation. KLe, EA, and XL performed the methodology. EA, MP, and XL gathered resources. MP, EA, AH, KLa, XL, MR, MS, and MH-F contributed in the writing—review and editing. All authors contributed to the article and approved the submitted version.

## Author Disclaimer

The views expressed in this article are those of the authors and do not necessarily reflect the official policies of the Department of Health and Human Services; nor does any mention of trade names, commercial practices, or organization imply endorsement by the United States Government.

## Conflict of Interest

The authors declare that the research was conducted in the absence of any commercial or financial relationships that could be construed as a potential conflict of interest.

## Publisher’s Note

All claims expressed in this article are solely those of the authors and do not necessarily represent those of their affiliated organizations, or those of the publisher, the editors and the reviewers. Any product that may be evaluated in this article, or claim that may be made by its manufacturer, is not guaranteed or endorsed by the publisher.

## Footnotes

1
https://pubmlst.org/
